# Inequalities in health complaints: 20-year trends among adolescents in Scotland, 1998–2018

**DOI:** 10.3389/fpsyg.2023.1095117

**Published:** 2023-03-20

**Authors:** Joanna C. Inchley, Malachi Willis, Judith Mabelis, Judith Brown, Dorothy B. Currie

**Affiliations:** ^1^MRC/CSO Social and Public Health Sciences Unit, School of Health and Wellbeing, University of Glasgow, Glasgow, United Kingdom; ^2^School of Medicine, University of St Andrews, St Andrews, United Kingdom

**Keywords:** adolescents, mental health, health complaints, social inequalities, school survey, HBSC

## Abstract

This study examined trends in inequalities in health complaints among early adolescents in Scotland from 1998 to 2018. We analysed data from the Health Behaviour in School-aged Children (HBSC) survey conducted in Scotland in 1998, 2002, 2006, 2010, 2014 and 2018. A self-report questionnaire was administered in schools to a nationally representative sample of 11-, 13-, and 15-year-olds (*n* = 29,250). Health complaints were measured using a scale comprising four psychological symptoms (feeling low, feeling nervous, irritability and sleep difficulties) and four somatic symptoms (headache, backache, stomachache and dizziness). Socio-economic status was measured using the Family Affluence Scale. Between 1998 and 2018, there were significant increases in the proportion of girls and boys reporting feeling low, feeling nervous, sleep difficulties and backache. Prevalence of the eight individual health complaints was higher among girls and adolescents from lower affluence families. Socio-economic inequalities increased over time, such that declines in mental health were greatest among low affluence adolescents. The data show worsening trends in health complaints among Scottish adolescents between 1998 and 2018, particularly for girls and adolescents from low affluence families. Increasing inequalities in mental health highlight the need to address the underlying social and structural determinants of adolescent mental health.

## Introduction

Mental health conditions such as anxiety, depression and behavioural disorders are major causes of ill health among young people [World Health Organization (WHO), 2022]. There is widespread concern about worsening mental health among the adolescent population, with evidence of declining mental wellbeing in recent years. The State of the World’s Children Report (United Nations Children’s Fund [UNICEF], 2021) estimates that 16% of adolescents in Europe have a mental health condition such as depression, anxiety, eating disorders and attention deficit hyperactivity disorder. Similarly, recent data from the UK indicate that one in six young people aged 6–16 years has a probable mental disorder, an increase from one in nine in 2017 (Newlove-Delgado et al., 2021). A review of secular trends in population prevalence of child and adolescent mental health problems in the UK found that clinical diagnosis and treatment of child and adolescent psychiatric disorders had increased in recent decades and that substantial increases in emotional and behavioural problems were evident (Collishaw, 2015). More recently, a large-scale community-based survey of adolescents in England reported that two-fifths of adolescents aged 11–14 years scored above the abnormal threshold for emotional problems, conduct problems or hyperactivity (Deighton et al., 2019). Longitudinal studies have also shown increases in depressive symptoms (Patalay and Gage, 2019), and a study in five high-income countries found evidence of increasing antidepressant use among children and adolescents between 2005 and 2012, with highest rates in Denmark, Germany and the UK (Bachmann et al., 2016). A more recent study reported that antidepressant prescribing has continued to increase among 12–17-year-olds in England (Jack et al., 2020).

Similar trends have been found in other high-income countries (Bor et al., 2014; Cosma et al., 2020), suggesting that this may be, in part, a result of broader cultural shifts affecting adolescent wellbeing. For example, health complaints have increased among Italian adolescents between 2010 and 2018 with the strongest effect for psychological symptoms among 15-year-olds (Bersia et al., 2022). Potrebny et al. (2019) reported a similar increase among Norwegian adolescents, with increases in psychological complaints more evident among older adolescent girls compared with boys and younger adolescents. In Sweden, there was an increase in frequent and co-occurring psychosomatic symptoms among 15-year-olds from 1985 to 2017, with higher prevalence amongst girls (Högberg et al., 2022).

Identifying groups of young people who may be at greater risk of experiencing mental health problems is important for targeting interventions and resources effectively. As well as evidence of clear age and gender differences emerging from recent studies, there are clear socio-economic inequalities in mental health: young people from more deprived backgrounds are more likely to experience poor subjective wellbeing and mental ill health. For example, a systematic review published in 2013 found that children and adolescents from more socioeconomically disadvantaged backgrounds were two to three times more likely to develop mental health problems (Reiss, 2013). Prevalence of emotional problems, conduct problems and hyperactivity among early adolescents in England was higher among those eligible for free school meals (Deighton et al., 2019). Furthermore, higher prevalence of adolescent anxiety and depression have been found to be associated with living in a single-parent household (Hafstad et al., 2021), and maternal education at birth is associated with socio-emotional problems at age 14 (Straatmann et al., 2019). Findings from the cross-national Health Behaviour in School-aged Children (HBSC) study show fairly consistent inequalities in mental health across countries; adolescents from higher affluence families report higher life satisfaction, better self-rated health and lower levels of multiple health complaints (Inchley et al., 2020a). International findings from this study have shown similar patterns (e.g., Hammami et al., 2022) and suggest that inequalities in subjective health have widened in recent years (Elgar et al., 2015; Chzhen et al., 2016).

Adolescence is a critical development period during which many mental health issues emerge, with the majority of long-term mental health conditions developing before the age of 24 (Blakemore, 2019). Poor mental health in adolescence can have a major impact on young people’s lives, affecting their social relationships, experiences at school, as well as longer term health and educational outcomes. Therefore, identifying mental health concerns at an early age and providing access to appropriate support and services are particularly important. However, evidence suggests that mental health services are stretched and many young people are not able to access the services they need. For many, the situation appears to have been exacerbated by the COVID-19 pandemic and particularly for those adolescents who were already vulnerable (Samji et al., 2022). School closures and lockdown measures stripped young people of the social structures and support that are so critical for healthy development during the adolescent years. Access to outside spaces and physical activity opportunities were also limited, and many recreational activities stopped. While not all young people experienced the impact of the pandemic in the same way, adverse effects on mental wellbeing, feelings of loneliness and sleep were found, particularly for young people with pre-existing vulnerabilities (e.g., Mansfield et al., 2021; Thakur et al., 2022; Tyack et al., 2022).

Monitoring long-term trends in mental health is important for understanding changing prevalence in disease burden on young people over time, identifying priorities for action and monitoring the impact of national policies and programmes as well as social changes. However, the ability to track mental health trends has been undermined by a lack of long-term studies that use comparable measures over time. The HBSC study provides data on early adolescents from the 1990s, thus providing a unique data source to investigate secular changes in mental health and other health outcomes over time. Using data from the HBSC Scotland survey, we investigated changes in psychological and somatic health complaints as an indicator of mental health over a 20-year period from 1998 to 2018. Somatic complaints are included because there is evidence that mental health problems in children and adolescents may initially present as somatic symptoms (Shapiro and Nguyen, 2010). Other studies amongst adolescents indicate an association between the number of somatic symptoms and poorer mental health (Ando et al., 2013; Högberg et al., 2022). Further, we examined the extent to which trends differ by gender and socio-economic status. Specifically, we aimed for to address the following research questions: (1) Did the prevalence of multiple health complaints and individual psychological or somatic symptoms among 11–15-year-olds in Scotland change between 1998 and 2018? and (2) Did the rate of change vary by gender or family affluence?

## Method

### Procedure

The Health Behaviour in School-aged Children (HBSC) study is a World Health Organization Collaborative Cross-National Survey, conducted every four years in member countries. The HBSC survey collects self-reported data on the health and wellbeing of adolescents and the social context in which they grow up. The survey is administered in schools to a nationally representative sample of 11-, 13-, and 15-year-olds with students completing the questionnaire anonymously in a classroom setting. Scotland joined the HBSC Study in 1986 and has conducted national surveys since 1990. In each survey year, the class is the primary sampling unit, stratified by school grade, and the sample is proportionally stratified by school funding (Local Education Authority (LEA) funded or independent) and by education authority (for LEA funded schools). Ethical approval for each survey round was granted at institutional level by the University hosting the national HBSC team.

### Participants

We analysed data from the Scottish HBSC surveys from 1998 to 2018. Because our primary research question focused on the effects of gender and family affluence, we semi-randomly selected a subsample of students from each year to ensure that our findings were not confounded by small differences in the distribution of gender and our measure of socio-economic position (the Family Affluence Scale) across survey years. The final dataset fixed the distribution of the three-category family affluence measure in the samples such that 20% of girls and 20% of boys within a year were in the low FAS group, 60% of each were in the medium FAS group, and 20% of each were in the high FAS group (see Supplementary Table 1); further, because FAS scores are relative to gender, we ensured equivalent numbers of girls and boys within each survey year. This restricted dataset provided greater confidence that any trends over time were not due to varying distributions of family affluence across survey year. The resulting subsample comprised 29,250 students: 1998 (*n* = 5,140), 2002 (*n* = 2,390), 2006 (*n* = 5,520), 2010 (*n* = 4,010), 2014 (*n* = 8,330), and 2018 (*n* = 3,860). Of the overall analytic sample, 49.9% were girls, 34.6% of students were in Primary 7 (11-year-olds), 34.0% in Secondary 2 (13-year-olds), and 31.4% in Secondary 4 (15-year-olds).

### Measures

#### Health complaints

The HBSC Symptom Checklist (HBSC-SCL) was used to measure students’ subjective health complaints. Health complaints are self-reported health symptoms which provide an indicator of mental health. Symptoms commonly co-occur and may be experienced by individuals with or without a specific diagnosis, reflecting both everyday experiences and health problems (Haugland and Wold, 2001). The HBSC-SCL is a non-clinical measure comprising eight items across two domains: psychological (i.e., feeling low, irritability, feeling nervous, sleeping difficulties) and somatic (i.e., headache, stomach-ache, backache, dizziness), which has been shown to be valid and reliable within adolescent populations (Haugland and Wold, 2001; Ravens-Sieberer et al., 2009; Heinz et al., 2022). Students reported how often they experienced each of the eight symptoms over the last 6 months: “about every day,” “more than once a week,” “about every week,” “about every month” and “rarely or never.” We defined multiple health complaints as experiencing two or more of the eight symptoms at least once a week (Ravens-Sieberer et al., 2009), which is an approach that can aid practical interpretation of the findings and has been used in international publications (e.g., Hammami et al., 2022). Further, we examined the individual psychological and somatic symptoms.

#### Family affluence

The Family Affluence Scale (FAS) is a composite measure of material wealth. FAS is a useful socioeconomic proxy for family wealth in youth surveys where parental responses about income or wealth are unavailable. Across three decades, FAS has been revised to reflect changing historical and technological conditions. The first version of the scale, FAS I, included family cars, child having a separate bedroom, and telephone ownership (Currie et al., 1997). For the 2002 HBSC survey, FAS II was created to include number of computers in the family and holidays abroad, and removing telephone ownership (Currie et al., 2008). For the 2014 HBSC survey, an extensive validation study led to the development of the six-item FAS III: car ownership, own bedroom, holidays abroad, number of computers, dishwasher, and number of bathrooms (Torsheim et al., 2016). To compare scores across these three versions of FAS used from 1998 to 2018, we used summed scores to create indices of relative ranks within survey year, gender, and age group, using ridit transformations (Elgar et al., 2017). Students were categorized into three categories based on their ranked scores: low FAS (bottom 20%), medium FAS (middle 60%), and high FAS (upper 20%). Because of the limited number of FAS categories, perfect 20/60/20 splits are not always obtained. As described under participants above, to simplify interpretation of trends over time a semi-random subsample was drawn for each year matching the desired 20/60/20 distribution of the three affluence categories (see Supplementary Table 1).

### Analysis

We used Stata’s (version 16.1) complex survey analysis functions to incorporate weighting and survey design. Post-stratification weights were applied to surveys from 2010 onwards to make the sample representative of Scottish Primary 7, Secondary 2 and Secondary 4 pupils with respect to relative representation between Local Education Authorities (geographic strata), school funding (state or independent), school denomination status (non-denominational or denominational) and rurality of school (based on Scottish Government rural–urban 6-point classification). To test our research questions, we conducted hierarchical binary logistic regression models with multiple health complaints as the outcome. In the first step of the model (RQ1), predictor variables included survey year (continuous), gender (dichotomous), and family affluence (trichotomous) with school grade (trichotomous) as a covariate. In the second step (RQ2), we included interaction terms testing whether the effect of survey year was moderated by gender or family affluence. We provided 95% confidence intervals for odds ratios and indicated whether they were statistically significant (α < 0.05).

## Results

### Multiple health complaints

**Figure 1:**
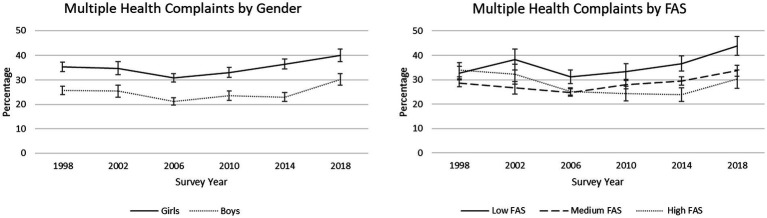
Trends in percentages of reported multiple health complaints (i.e., at least two of eight weekly symptoms), by gender and by Family Affluence Scale (FAS). 95% confidence intervals are depicted.

The percentage of students reporting multiple health complaints in surveys ranged from 26.1% in 2006 to 35.0% in 2018 (Table 1), with a small but significant increase on average from 1998 to 2018. Controlling for school grade, girls were more likely than boys to report at least two symptoms more than once a week, and low FAS students had greater odds of reporting multiple health complaints than both medium and high FAS students (Table 2). Further, trends over time significantly differed between low FAS and high FAS students. As depicted in Figure 1, this significant interaction was characterized by a generally increasing percentage of low FAS students reporting at least two symptoms more than once a week from 1998 to 2018 compared with slight decreases for high FAS students during this period.

**Table 1:** Percentages of multiple health complaints, somatic symptoms, and psychological symptoms, by survey year.

	1998	2002	2006	2010	2014	2018
Multiple health complaints						
At least 2 of 8 weekly	30.6	30.1	26.1	28.3	29.8	35.0
95% CI	(29.3–31.8)	(28.3–32.0)	(24.9–27.3)	(26.9–29.8)	(28.5–31.2)	(33.3–36.8)
Psychological Symptoms						
Feel low	10.5	12.4	10.8	13.5	16.5	19.0
95% CI	(9.7–11.4)	(11.1–13.8)	(10.0–11.7)	(12.5–14.6)	(15.4–17.6)	(17.6–20.4)
Irritable	24.8	24.0	22.0	22.9	22.9	23.5
95% CI	(23.6–26.0)	(22.3–25.8)	(20.9–23.1)	(21.6–24.3)	(21.6–24.1)	(22.0–25.1)
Nervous	14.9	15.6	12.1	15.2	19.6	24.4
95% CI	(14.0–16.0)	(14.2–17.1)	(11.2–13.0)	(14.1–16.4)	(18.5–20.8)	(22.9–26.0)
Sleep difficulties	22.2	21.8	21.7	24.3	23.1	30.2
95% CI	(21.1–23.4)	(20.2–23.5)	(20.7–22.9)	(22.9–25.6)	(21.9–24.4)	(28.6–31.9)
Somatic Symptoms						
Headache	18.2	16.1	13.0	15.4	14.9	16.1
95% CI	(17.2–19.3)	(14.7–17.6)	(12.1–13.9)	(14.3–16.6)	(13.9–16.0)	(14.8–17.4)
Stomachache	11.3	9.9	7.8	9.1	8.9	9.1
95% CI	(10.4–12.2)	(8.7–11.1)	(7.1–8.5)	(8.2–10.0)	(8.1–9.8)	(8.0–10.2)
Backache	7.9	6.5	6.8	9.3	8.8	10.4
95% CI	(7.2–8.7)	(5.6–7.6)	(6.2–7.5)	(8.5–10.3)	(8.0–9.7)	(9.3–11.6)
Dizzy	11.4	11.0	10.1	11.3	10.7	12.7
95% CI	(10.6–12.3)	(9.8–12.4)	(9.3–10.9)	(10.4–12.4)	(9.8–11.6)	(11.6–14.0)
Denominator	5,125	2,390	5,469	3,979	8,267	3,829

**Table 2:** Odds ratios of reporting multiple health complaints (i.e., at least two of eight weekly symptoms).

	OR	95% CI	*p*
Step 1
Survey year	1.01	1.00–1.01	<0.001
Sex (ref = Boy)
Girl	1.69	1.59–1.79	<0.001
FAS (ref = Low)
Medium	0.74	0.69–0.79	<0.001
High	0.70	0.64–0.76	<0.001
Grade (ref = P7)
S2	1.37	1.27–1.47	<0.001
S4	1.67	1.55–1.80	<0.001
Step 2
Survey year*Sex
Girl	1.01	1.00–1.02	0.164
Survey year*FAS
Medium	0.99	0.98–1.00	0.059
High	0.96	0.95–0.98	<0.001

FAS, Family Affluence Scale; P7, primary level 7 (11-year-olds); S2, secondary level 2 (13-year-olds); S4, secondary level 4 (15-year-olds).

### Psychological symptoms

**Figure 2:**
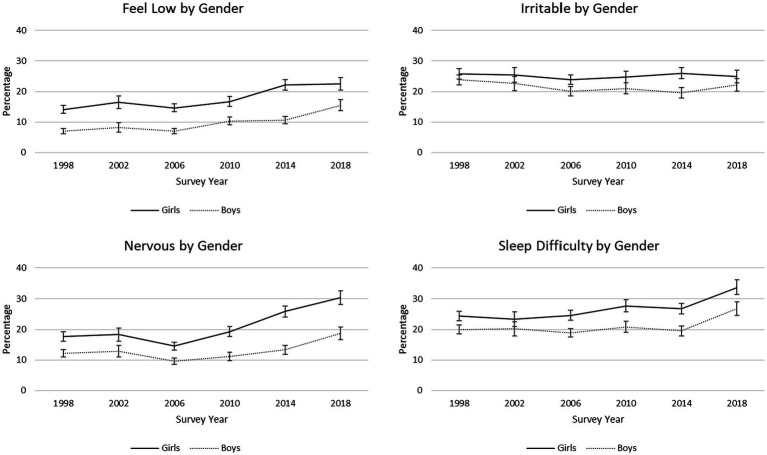
Trends in percentages of reported psychological symptoms, by gender. 95% confidence intervals are depicted.

**Figure 3:**
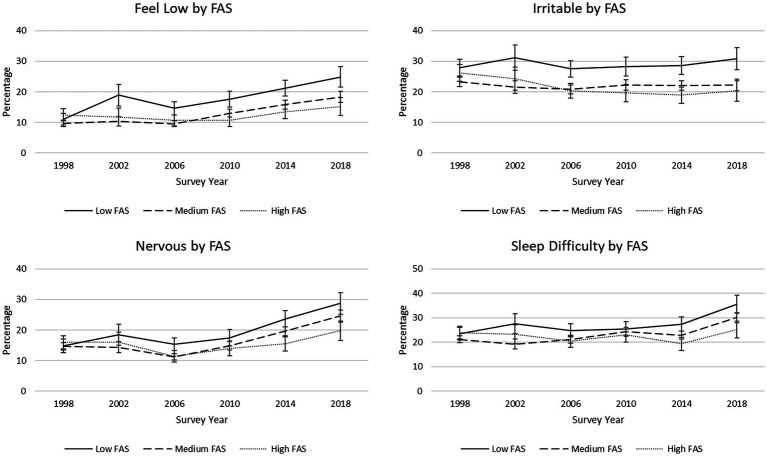
Trends in percentages of reported psychological symptoms, by Family Affluence Scale (FAS). 95% confidence intervals are depicted.

Descriptive statistics are presented by survey year for individual psychological and somatic symptoms (Table 1). On average, prevalence of three of the four psychological symptoms—feeling low, feeling nervous, and sleep difficulties—significantly increased from 1998 to 2018. Reports of feeling low changed the most over time; there was a 3.9% annual increase in the percentage of students reporting this symptom when controlling for sex, FAS, and grade. During this period, girls were more likely than boys to report experiencing each of the four psychological symptoms more than once a week (Table 3), and the percentage of girls reporting feeling nervous increased at a greater rate from 1998 to 2018 than it did for boys (Figure 2). Low FAS students had greater odds of reporting each psychological complaint than high FAS students. Further, trends over time significantly differed between low FAS and high FAS students for all four psychological symptoms. For feeling low, feeling nervous, and sleep difficulties, this significant interaction was characterized by the percentage of low FAS students reporting each symptom increasing at a greater rate from 1998 to 2018 than the increases in high FAS students during this period (Figure 3). However, the percentage of low FAS students reporting feeling irritable was generally stable from 1998 to 2018 while fewer high FAS students reported this symptom over time.

**Table 3:** Odds ratios of reporting weekly psychological symptoms.

	Feel Low			Irritable			Nervous			Sleep Difficulty		
	OR	95% CI	*p*	OR	95% CI	*p*	OR	95% CI	*p*	OR	95% CI	*p*
Step 1
Survey year	1.04	1.03–1.05	<0.001	1.00	0.99–1.00	0.142	1.03	1.03–1.04	<0.001	1.02	1.01–1.02	<0.001
Sex (ref = Boy)
Girl	2.10	1.94–2.28	<0.001	1.25	1.18–1.33	<0.001	1.87	1.73–2.01	<0.001	1.40	1.21–1.49	<0.001
FAS (ref = Low)												
Medium	0.70	0.64–0.77	<0.001	0.72	0.67–0.78	<0.001	0.82	0.75–0.89	<0.001	0.82	0.76–0.88	<0.001
High	0.66	0.59–0.75	<0.001	0.68	0.62–0.75	<0.001	0.73	0.64–0.81	<0.001	0.76	0.69–0.84	<0.001
Grade (ref = P7)
S2	1.70	1.51–1.87	<0.001	1.54	1.42–1.67	<0.001	1.33	1.22–1.46	<0.001	0.92	0.85–0.99	0.026
S4	2.26	2.04–2.50	<0.001	1.71	1.57–1.85	<0.001	1.53	1.40–1.68	<0.001	1.11	1.03–1.20	0.006
Step 2
Survey year*Sex
Girl	0.99	0.98–1.00	0.189	1.01	1.00–1.02	0.033	1.02	1.01–1.03	<0.001	1.01	1.00–1.02	0.124
Survey year*FAS
Medium	0.99	0.98–1.00	0.189	0.99	0.98–1.00	0.115	0.99	0.98–1.00	0.173	1.00	0.99–1.01	0.682
High	0.97	0.95–0.98	<0.001	0.97	0.96–0.99	<0.001	0.97	0.95–0.98	<0.001	0.97	0.96–0.99	<0.001

FAS, Family Affluence Scale. P7, primary level 7 (11-year-olds). S2, secondary level 2 (13-year-olds). S4, secondary level 4 (15-year-olds).

### Somatic symptoms

**Figure 4:**
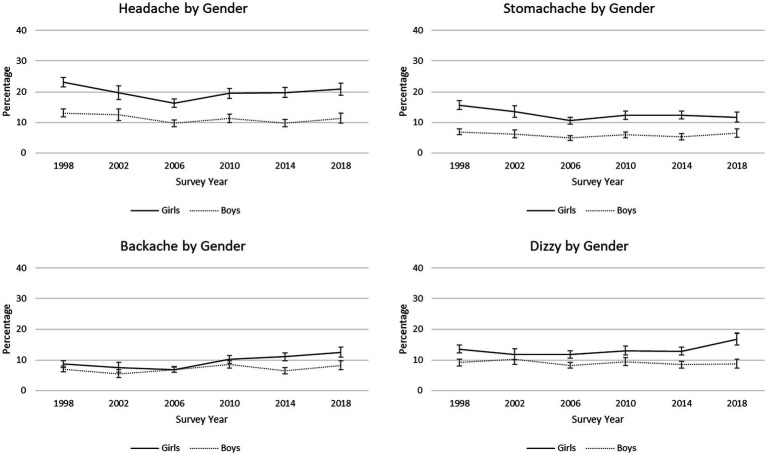
Trends in percentages of reported somatic symptoms, by gender. 95% confidence intervals are depicted.

**Figure 5:**
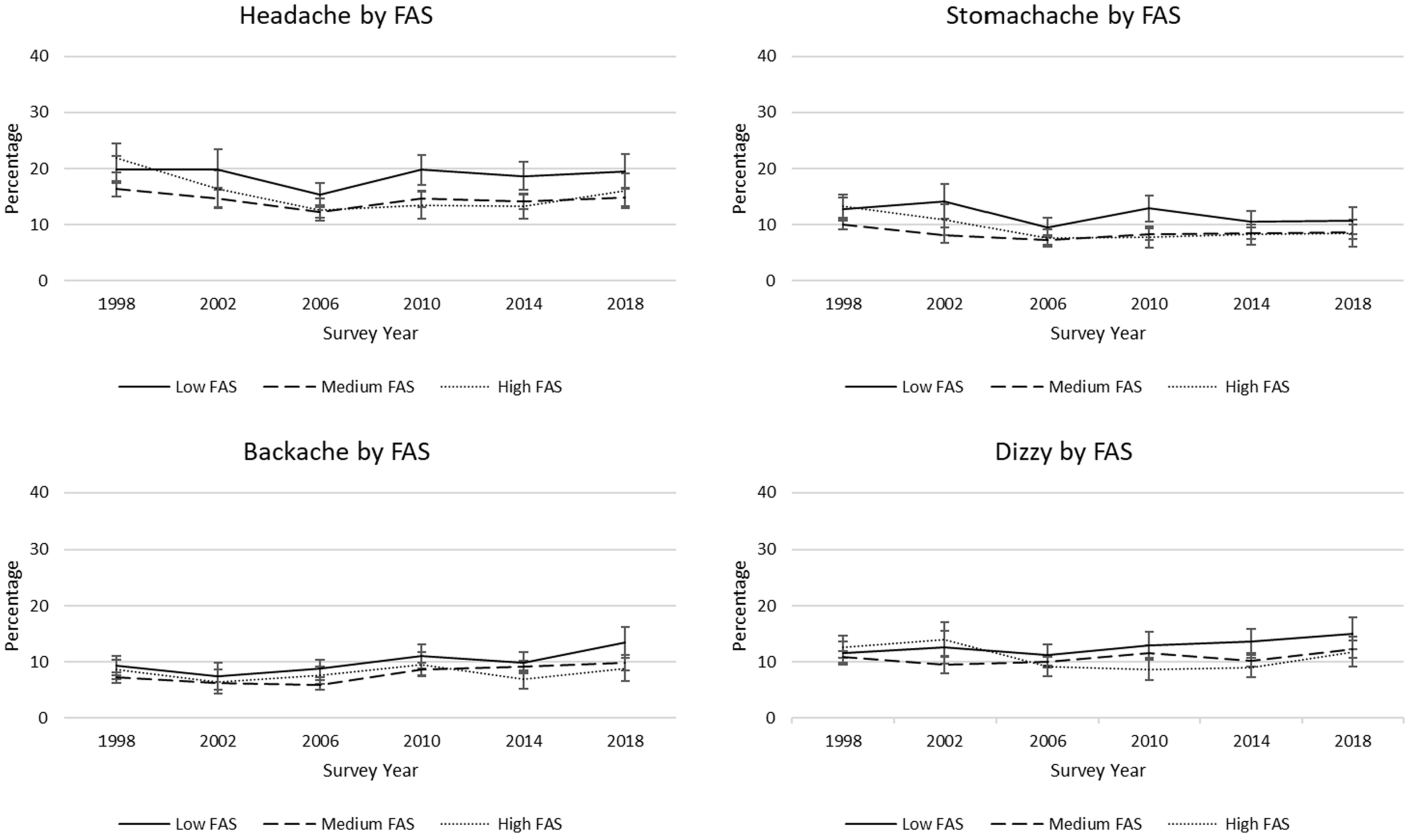
Trends in percentages of reported somatic symptoms, by Family Affluence Scale (FAS). 95% confidence intervals are depicted.

On average, students’ reports of backache significantly increased from 1998 to 2018 but prevalence of headache and stomachache significantly decreased during this period. Reported backache changed the most over time; there was a 2.0% annual increase in the percentage of students reporting this symptom when controlling for sex, FAS, and grade. Girls were more likely than boys to report experiencing each of the four somatic symptoms more than once a week (Table 4), and the percentage of girls reporting backaches and dizziness increased from 1998 to 2018 but remained relatively stable for boys (Figure 4). Low FAS students had greater odds of reporting each somatic complaint than both medium and high FAS students. Further, trends over time significantly differed between low FAS and high FAS students for all four somatic symptoms. This significant interaction was characterized by the percentage of low FAS students reporting backaches and dizziness increasing from 1998 to 2018 while prevalence of these symptoms remained generally stable for high FAS students during this period (Figure 5). For headaches, the percentage of low FAS students was stable from 1998 to 2018 while fewer high FAS students reported this symptom over time. Finally, the percentage of low FAS students reporting stomach-ache decreased at a slightly slower rate than it did for high FAS students.

**Table 4:** Odds ratios of reporting weekly somatic symptoms.

	Headache			Stomachache			Backache			Dizzy		
	OR	95% CI	*p*	OR	95% CI	*p*	OR	95% CI	*p*	OR	95% CI	*p*
Step 1
Survey year	0.99	0.99–1.00	0.013	0.99	0.98–1.00	0.001	1.02	1.01–1.03	<0.001	1.00	1.00–1.01	0.243
Sex (ref = Boy)
Girl	2.00	1.86–2.16	<0.001	2.34	2.23–2.58	<0.001	1.39	1.26–1.53	<0.001	1.57	1.44–1.71	<0.001
FAS (ref = Low)
Medium	0.76	0.69–0.83	<0.001	0.72	0.65–0.81	<0.001	0.82	0.73–0.92	<0.001	0.83	0.75–0.92	<0.001
High	0.81	0.72–0.90	<0.001	0.79	0.69–0.90	<0.001	0.81	0.70–0.93	0.004	0.80	0.70–0.91	<0.001
Grade (ref = P7)
S2	1.46	1.32–1.61	<0.001	1.11	0.99–1.24	0.073	1.66	1.45–1.90	<0.001	1.30	1.17–1.44	<0.001
S4	1.83	1.67–2.01	<0.001	1.10	0.98–1.23	0.096	2.76	2.43–3.13	<0.001	1.43	1.29–1.59	<0.001
Step 2
Survey year*Sex
Girl	1.01	1.00–1.02	0.159	0.99	0.98–1.01	0.375	1.02	1.01–1.04	0.004	1.01	1.00–1.03	0.022
Survey year*FAS
Medium	0.99	0.98–1.00	0.125	1.01	0.99–1.02	0.551	1.00	0.98–1.01	0.667	0.99	0.97–1.00	0.087
High	0.97	0.96–0.99	0.002	0.98	0.96–1.00	0.101	0.98	0.98–1.00	0.046	0.97	0.95–0.99	0.001

FAS, Family Affluence Scale. P7, primary level 7 (11-year-olds). S2, secondary level 2 (13-year-olds). S4, secondary level 4 (15-year-olds).

## Discussion

This paper describes trends in health complaints among early adolescents in Scotland over the last two decades. In line with other recent studies, prevalence of health complaints was consistently higher among girls and among young people from less affluent families (Reiss, 2013; Bor et al., 2014). This was the case for the composite measure of health complaints as well as several individual symptoms. Prevalence was highest for psychological symptoms reflecting growing concerns about poor psychological wellbeing among adolescents, especially older adolescent girls. Changes over time were observed mainly for psychological symptoms with more marked increases in recent years. Since the late 1990s, the proportion of adolescents reporting that they regularly feel low has doubled; smaller but significant increases existed for feeling nervous and sleep difficulties. Even though increased negative mood is common during the early adolescent years these findings are concerning because this symptom can be a precursor to more serious psychological problems for some young people (Dietvorst et al., 2021). Similarly, sleep is essential for healthy development during the adolescent years and poor sleep is associated with impaired cognitive function, depressive symptoms and risk behaviours (Owens et al., 2014). Feeling nervous may reflect higher levels of anxiety or lower levels of confidence, both of which can affect young people’s ability to function well across a range of domains including school and social situations. Fewer changes over time were observed for somatic symptoms except for backache, for which prevalence fluctuated over time but appears to have increased since the mid-2000s among girls. Our finding of an increasing prevalence of backpain corresponds with a recent study from Denmark, which also found higher prevalence among adolescents from lower socio-economic families (Holstein et al., 2022).

There was clear evidence of widening inequalities in mental health in that the gap between low affluence adolescents and their high affluence peers increased for each of the eight health complaints, particularly in more recent years. Sharper increases in several psychological and somatic symptoms were observed among lower affluent adolescents. Further, while prevalence of irritability and headaches remained relatively stable over time for low affluent adolescents, these complaints decreased among higher affluence adolescents across this same period. A decline in reported stomachache was observed across the sample on average but was less marked among low affluence individuals. These trends reinforce previous evidence that the burden of poor mental wellbeing disproportionately affects young people from more disadvantaged backgrounds, and that this disparity between young people from different socio-economic backgrounds has worsened in recent years.

A range of structural and social factors may be perpetuating these trends and contributing to discrepancies in mental health risk and outcomes. Increases in academic pressure, social media use, economic instability and family breakdown have all been proposed as potential drivers (Gunnell et al., 2018). One of the main explanatory theories is the social causation hypothesis which focuses on differential exposure to stressful life events associated with deprivation (Reiss, 2013). Young people growing up in more disadvantaged circumstances are more likely to experience stressors across a range of domains (e.g., financial, social and educational) and may also have less access to important resources or buffers such as social support and health or community-based services. Recent studies among European adolescents, for example, have found that negative life events are associated with higher prevalence of mental health problems (Reiss et al., 2019) and that exposure to negative life events and family stressors explains some of the association between low socio-economic status and poor mental health (Bøe et al., 2018).

In relation to school, the educational stressors hypothesis states that mental health problems are linked to stressors within the school context (Högberg, 2021). Greater focus on educational attainment within knowledge economies may raise academic expectations and increase the pressure on adolescents to perform well at school. Several studies have demonstrated a relationship between schoolwork pressure and poor mental health. For example, Cosma et al. (2020) examined cross-national trends in mental wellbeing and found that schoolwork pressure was associated with more frequent psychosomatic health complaints and partly explained increases in psychosomatic complaints over time. Data from the HBSC study also show that levels of perceived schoolwork pressure are particularly high among Scottish 15-year-olds, with 74% of girls and 53% of boys reporting a lot or some pressure from schoolwork, and similar findings observed for other UK countries (Inchley et al., 2020a). Furthermore, levels of perceived schoolwork pressure have increased in Scotland since 1994 (Inchley et al., 2020b) and, in 2018, were much higher among boys from low affluence families compared to those from high affluence families whereas there was no significant difference among girls (Inchley et al., 2020a). Schools have a key role in promoting and supporting the mental health of adolescents and have the potential to address inequalities in health by reaching the whole school population, although evidence on the extent to which this occurs in practice is mixed (Moore et al., 2015). Our findings suggest that adolescents may be more vulnerable to mental health problems at particular stages of their school career, such as following the transition to secondary school and the exam years, likely reflecting a combination of biological, psychological and social influences during this important developmental and transitional period.

Early adolescence is a critical time for early intervention to prevent longer-term mental health problems and there is an urgent need for more investment in mental health services and support to meet increasing demand, particularly targeted at young people from more disadvantaged backgrounds who may, not only have greater vulnerability and exposure to risks, but also be less able to access available support. Schools are increasingly on the frontline dealing with mental health crises on a day-to-day basis, but with very limited resources. Again, there is a need for support and training for school staff to enable them to meet the mental health needs of their school communities more effectively, as well as effective referral pathways on to more specialist support for those young people that need it.

A major strength of this study is the ability to draw on unique 20-year data from six consecutive waves of the HBSC Scotland survey using the same survey design items to maximise comparability over time. Some caution should be applied to interpretation of the trends as we have no information on the intervening years and the data are cross-sectional. We used the HBSC health complaints scale as an indicator of mental health. This is a well-used and validated measure internationally. However, mental health is a complex construct and other mental health indicators might show different patterns by gender and socio-economic status over time.

In conclusion, our findings highlight important gender and socio-economic inequalities in mental health over the past 20 years in Scotland. Overall, prevalence of psychosomatic symptoms has increased reflecting worsening mental health among the adolescent population. These increases are not equally distributed across the population, with young people from less affluent backgrounds having experienced more marked increases, particularly in relation to low mood, nervousness and sleep difficulties. That such inequalities have persisted for two decades demonstrates the need for further investment and action to address the underlying social and structural determinants of mental health within the adolescent population, alongside more universal prevention strategies. Ongoing monitoring is also important, particularly in the context of the COVID-19 pandemic, to assess its longer-term impact among adolescents and particularly among those from lower socioeconomic backgrounds who may be more at risk of developing mental health problems.

## Data availability statement

Publicly available datasets were analyzed in this study. This data can be found here: https://www.uib.no/en/hbscdata/113290/open-access.

## Ethics statement

The studies involving human participants were reviewed and approved by the University of St Andrews Medical School Teaching and Research Ethics Committee (2014 and 2018), University of Edinburgh School of Education Ethics Committee (2002, 2006, 2010), and University of Edinburgh Medical School Ethics Committee (1998). Written informed consent from the participants’ legal guardian/next of kin was not required to participate in this study in accordance with the national legislation and the institutional requirements.

## Author contributions

MW led the data analysis. JI led on writing of the manuscript. DC, JM, and JB contributed to drafting of the manuscript and final review. All authors conceived and designed the study.

## Funding

Funding for the Scottish HBSC Survey was provided by Public Health Scotland (previously, NHS Health Scotland). This work also was supported by the Medical Research Council (MC_UU_00022/1) and the Chief Scientist Office of the Scottish Government Health and Social Care Directorate (SPHSU16). The opinions expressed in this paper are those of the authors and do not necessarily reflect those of Public Health Scotland as commissioners of the work or the University Court of the University of Glasgow as providers of the work.

## Conflict of interest

The authors declare that the research was conducted in the absence of any commercial or financial relationships that could be construed as a potential conflict of interest.

## Publisher’s note

All claims expressed in this article are solely those of the authors and do not necessarily represent those of their affiliated organizations, or those of the publisher, the editors and the reviewers. Any product that may be evaluated in this article, or claim that may be made by its manufacturer, is not guaranteed or endorsed by the publisher.
