# Supplementary material for: Inequalities in health complaints: 20-year trends among adolescents in Scotland, 1998–2018
Source: Front Psychol. 2023 Mar 20;14:1095117. doi: 10.3389/fpsyg.2023.1095117 (PMC10067662; doi:10.3389/fpsyg.2023.1095117)
Supplement: Supplementary file 1 [file Table_1.docx]

**Supplementary Table 1**

Original Sample Versus Semi-Random Subsample Selected According to Family Affluence Scale (FAS)

|  |  | Total | Girls | | | Boys | | |
| --- | --- | --- | --- | --- | --- | --- | --- | --- |
|  |  |  | Low FAS | Medium FAS | High FAS | Low FAS | Medium FAS | High FAS |
| 1998 | Original Sample | 5631 | 576 (20.1%) | 1720 (60.2%) | 562 (19.7%) | 601 (21.7%) | 1658 (59.8%) | 514 (18.5%) |
|  | Random Subsample | 5140 | 514 (20%) | 1542 (60%) | 514 (20%) | 514 (20%) | 1542 (60%) | 514 (20%) |
| 2002 | Original Sample | 4404 | 577 (26.7%) | 1342 (62.2%) | 239 (11.1%) | 562 (25.0%) | 1331 (59.3%) | 353 (15.7%) |
|  | Random Subsample | 2390 | 239 (20%) | 717 (60%) | 239 (20%) | 239 (20%) | 717 (60%) | 239 (20%) |
| 2006 | Original Sample | 6149 | 617 (20.0%) | 1917 (62.1%) | 552 (17.9%) | 706 (23.0%) | 1718 (56.1%) | 639 (20.9%) |
|  | Random Subsample | 5520 | 552 (20%) | 1656 (60%) | 552 (20%) | 552 (20%) | 1656 (60%) | 552 (20%) |
| 2010 | Original Sample | 6771 | 401 (11.7%) | 2103 (61.3%) | 928 (27.0%) | 508 (15.2%) | 1917 (57.4%) | 914 (27.4%) |
|  | Random Subsample | 4010 | 401 (20%) | 1203 (60%) | 401 (20%) | 401 (20%) | 1203 (60%) | 401 (20%) |
| 2014 | Original Sample | 10839 | 969 (18.0%) | 3529 (65.6%) | 880 (16.4%) | 833 (15.2%) | 3338 (61.1%) | 1290 (23.6%) |
|  | Random Subsample | 8330 | 833 (20%) | 2499 (60%) | 833 (20%) | 833 (20%) | 2499 (60%) | 833 (20%) |
| 2018 | Original Sample | 5286 | 425 (15.7%) | 1745 (64.5%) | 535 (19.8%) | 386 (15.0%) | 1582 (61.3%) | 613 (23.8%) |
|  | Random Subsample | 3860 | 386 (20%) | 1158 (60%) | 386 (20%) | 386 (20%) | 1158 (60%) | 386 (20%) |
